# Structural Insights into the Penicillin-Binding Protein 4 (DacB) from *Mycobacterium tuberculosis*

**DOI:** 10.3390/ijms25020983

**Published:** 2024-01-12

**Authors:** Sung-Min Kang, Do-Hee Kim

**Affiliations:** 1 College of Pharmacy, Duksung Women’s University, Seoul 01369, Republic of Korea; 2 Jeju Research Institute of Pharmaceutical Sciences, College of Pharmacy, Jeju National University, Jeju 63243, Republic of Korea; 3 Interdisciplinary Graduate Program in Advanced Convergence Technology & Science, Jeju National University, Jeju 63243, Republic of Korea

**Keywords:** *Mycobacterium tuberculosis*, antibiotics, penicillin-binding protein

## Abstract

*Mycobacterium tuberculosis*, a major cause of mortality from a single infectious agent, possesses a remarkable mycobacterial cell envelope. Penicillin-Binding Proteins (PBPs) are a family of bacterial enzymes involved in the biosynthesis of peptidoglycan. PBP4 (DacB) from *M. tuberculosis* (MtbPBP4) has been known to function as a carboxypeptidase, and the role and significance of carboxypeptidases as targets for anti-tuberculosis drugs or antibiotics have been extensively investigated over the past decade. However, their precise involvement remains incompletely understood. In this study, we employed predictive modeling and analyzed the three-dimensional structure of MtbPBP4. Interestingly, MtbPBP4 displayed a distinct domain structure compared to its homologs. Docking studies with meropenem verified the presence of active site residues conserved in PBPs. These findings establish a structural foundation for comprehending the molecular function of MtbPBP4 and offer a platform for the exploration of novel antibiotics.

## 1. Introduction

*Mycobacterium tuberculosis* is a dangerous bacterium responsible for tuberculosis (TB), which is a significant global health concern, especially in developing countries [1]. *M. tuberculosis* has a thick, lipid-rich cell wall, which acts as a barrier and restricts the entry of many drugs, including β-lactam antibiotics. This impermeable cell wall hampers the penetration of antibiotics into the bacterial cell, reducing their efficacy. Additionally, it is crucial to acknowledge the significant role of efflux pumps in contributing to the beta-lactam tolerance of *M. tuberculosis* [2,3]. Therefore, β-lactam antibiotics are not typically used as first-line drugs for TB treatment due to their inherent resistance to *M. tuberculosis*. As a result, standard β-lactam antibiotic treatment for TB involves a combination of drugs [4,5]. Recently, multidrug-resistant (MDR) TB has become a critical issue in the treatment of *M. tuberculosis* infections [6]. MDR-TB occurs when the bacterium becomes resistant to two of the most potent first-line anti-tuberculosis drugs: isoniazid and rifampicin [7]. Patients with MDR-TB can experience treatment failure, the prolonged transmission of drug-resistant strains, and increased mortality rates [8]. Moreover, the emergence of extensively drug-resistant (XDR)TB has further complicated the management of TB infections [9]. Consequently, there is an urgent need to discover new antibiotics to effectively combat multidrug-resistant tuberculosis [10].

In bacteria, the peptidoglycan layer is essential for maintaining the shape and stability of the bacterial cell wall [11]. Penicillin-Binding Proteins (PBPs) are a family of bacterial enzymes involved in the biosynthesis of peptidoglycan. PBPs catalyze the polymerization of the glycan strand (transglycosylation) and the formation of cross-links between glycan chains (transpeptidation). Additionally, certain PBPs hydrolyze the terminal D-alanine of stem pentapeptides (DD-carboxypeptidation) or the peptide bond that links two glycan strands (endopeptidation) [12,13]. PBPs are classified into two main categories based on their molecular weight: high-molecular-weight (HMW) PBPs and low-molecular-weight (LMW) PBPs [14]. Class A PBPs are HMW PBPs with molecular weights typically exceeding 100 kDa [15]. They are responsible for crosslinking peptidoglycans in the cell wall and exhibit both transglycosylase and transpeptidase activities [16]. Class B PBPs have an N-terminal domain of unknown function and a C-terminal domain containing transpeptidase activity [17]. They are also HMW PBPs but are smaller in size compared to Class A PBPs [18]. Class C PBPs are LMW PBPs, usually having molecular weights below 50 kDa [19]. Although not directly involved in cell wall synthesis, they possess transpeptidase activity and participate in the remodeling and maintenance of the cell wall [13].

In *M. tuberculosis*, there are seven putative PBPs, comprising DacB, DacB1, DacB2, PbpA, PbpB, PonA1, and PonA2 [20]. Rv3627c is PBP4 (DacB) from *M. tuberculosis* (MtbPBP4) belonging to the Class C PBP group [21]. MtbPBP4 exhibits the characteristics of a novel carboxypeptidase [22], such as specific serine-type carboxypeptidase activity, and it cleaves the cross-links involving terminal alanine [23]. The mode of action of β-lactam antibiotics on MtbPBP4 involves inhibiting its carboxypeptidase activity. This activity is crucial for cleaving peptide bonds in peptidoglycan precursors and assembling the bacterial cell wall. By covalently binding to the active site serine of MtbPBP4, β-lactam antibiotics disrupt cell wall synthesis, leading to defects in the peptidoglycan structure and, eventually, bacterial cell lysis. This inhibition of cell wall assembly contributes to the anti-bacterial effects of β-lactam antibiotics against *M. tuberculosis* [22,24].

In this study, we aimed to gain structural insights into MtbPBP4 through a combination of bioinformatics, size-exclusion chromatography (SEC), modeling, molecular docking, and molecular dynamics simulations. The sequence analysis based on bioinformatics indicates that MtbPBP4 belongs to the peptidase family S13 as a D-Ala-D-Ala carboxypeptidase C and has conserved active site residues. The SEC results verified the state of MtbPBP4 as a monomer, and three-dimensional structural modeling exhibited that MtbPBP4 contains the conserved penicillin-binding (PB) domain with distinct differences with its homologs. Additionally, molecular docking with meropenem confirmed the putative interaction residues of MtbPBP4. This information can provide valuable insights to aid in the design of specific inhibitors that effectively disrupt the activity of MtbPBP4, assisting the development of novel and potent anti-tuberculosis drugs to combat MDR-TB effectively.

## 2. Results and Discussion

### 2.1. Sequence Analysis

According to a sequence-based analysis using InterPro [25], a transmembrane region at the N-terminus of MtbPBP4 (residues 1–28) is predicted to be a signal peptide, suggesting its involvement in membrane targeting or secretion. The remaining region (residues 29–461) is expected to be located outside the membrane, indicating that PBP4 functions in the extracellular region. InterPro analysis identified this region as belonging to peptidase family S13, specifically the D-Ala-D-Ala carboxypeptidase C (InterPro000667, residues 73–459) family, which possesses the conserved motif SXXK as the active site residue. In a previous study by Sauvage et al., PBPs from selected bacteria were classified, and Rv3627c (MtbPBP4) was assigned to class C PBP4 [13]. Except for MtbPBP4, the three-dimensional structures of several PBP4 homologs have been determined, including PBP4 from *Escherichia coli* (PDB ID: 2EX2) [26], PBP4a from *Bacillus subtilis* (PDB ID: 1W5D) [27], R39 from Actinomycetes (PDB ID: 1W79) [28], and PBP4 from *Haemophilus influenzae* (PDB ID: 3A3D) [29].

**Figure 1:**
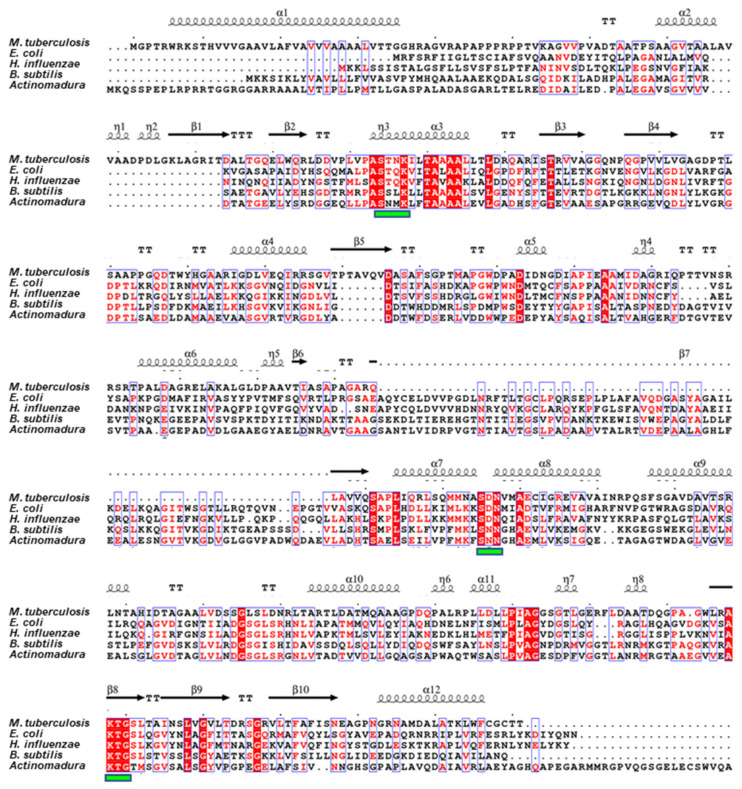
Sequence alignment of MtbPBP4 with its homologs. The alignment results of PBP4 from *M. tuberculosis* with PBP4 from *E. coli* (PDB ID: 2EX2), PBP4 from *H*. *influenzae* (PDB ID: 3A3D), PBP4a from *B*. *subtilis* (PDB ID: 1W5D), and R39 from Actinomycetes (PDB ID: 1W79) were analyzed using MultAlin [30] and ESPript 3.0 [31]. Conserved and similar residues are shown in red and blue boxes, respectively. The conserved motifs among PBP4s are marked by the green boxes under the sequences.

The sequence of MtbPBP4 was compared with other PBP4 homologs using MultAlin [30] and ESPript 3.0 [31], as depicted in Figure 1. In MtbPBP4, like its homologs, the SXXK motif is present, specifically Ser114-Thr115-Asn116-Lys117, which is conserved across PBP4 proteins [20]. Additionally, two other conserved motifs typical of LMW-PBPs were identified in MtbPBP4 as Ser295-Asp296-Asn297 (SXN) and Lys408-Thr409-Gly410 (KTG). These highly conserved residues are anticipated to play a critical role as a carboxypeptidase in the mycobacterial cell wall. However, PBP4 homologs exhibited a notable distinction in their sequence, which inserts approximately 70 amino acids near Arg241, adjacent to the β7 strand of MtbPBP4. The region anticipated to be the β7 in the secondary structure appears to deviate from a perfect β strand due to the lack of sufficient predictive data. This distinction in prediction results is a consequence of incomplete data in the prediction.

### 2.2. Oligomeric State of MtbPBP4 in Solution

**Figure 2:**
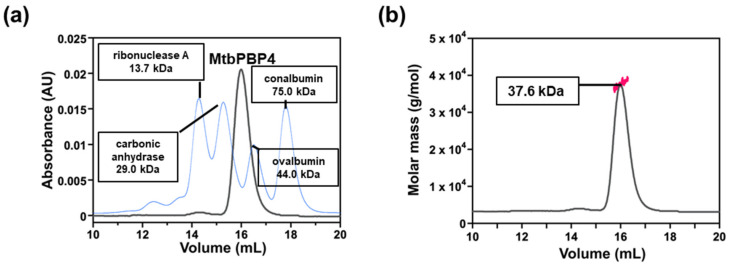
Oligomeric state analysis of MtbPBP4. (**a**) SEC chromatograms of MtbPBP4 and reference proteins and SEC-MALS chromatogram of MtbPBP4 showing the oligomeric state in solution. The UV absorption of MtbPBP4 (gray) and reference proteins (blue) at 280 nm are plotted as functions of the elution volume. (**b**) The calculated molecular mass in SEC-MALS is indicated as red dots. This result indicates that MtbPBP4 exists as a monomer in solution.

To verify the oligomeric state of MtbPBP4 in solution, we compared the elution time after gel filtration using four references (ribonuclease A 13.7 kDa, carbonic anhydrase 29.0 kDa, ovalbumin 44.0 kDa, and conalbumin 75.0 kDa) from the Gel Filtration Calibration kits (Cytiva, Marlborough, MA, USA). It was calculated that the elution time corresponded to a molecular weight of 36.2 kDa (Figure 2a). To further confirm this, an additional experiment using SEC-MALS was conducted, and the resulting peak corresponded in size to 37.6 kDa (Figure 2b). When considering the inclusion of the N-terminal 6xHis-tag, the theoretical molecular weight should be 42.3 kDa. However, our experimental results consistently indicated values of 37.6 kDa and 36.2 kDa. Despite a slight deviation from the theoretical value, this outcome confirms that MtbPBP4 primarily exists as a monomer in solution, and consequently, a monomer model was employed to construct a 3D model of MtbPBP4 to predict its structure. However, it should be acknowledged that only the soluble part of the protein was cloned and purified, so the transmembrane domain may contribute to a different oligomeric state of the protein.

### 2.3. Overall Structure of MtbPBP4

**Figure 3:**
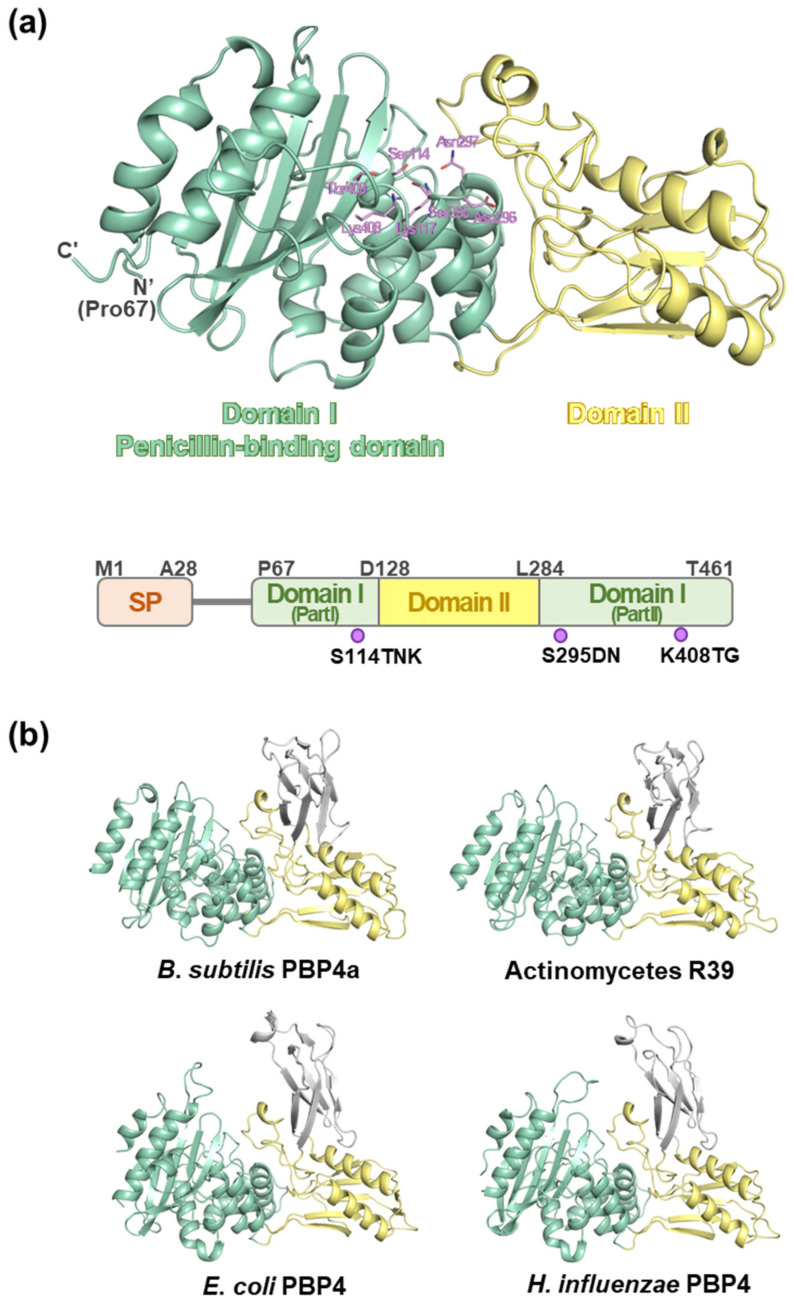
Overall predicted structure of MtbPBP4. (**a**) The overall predicted MtbPBP4 structure was divided into two domains: domain I, as the penicillin-binding domain, is colored in light green, and domain II is colored in light yellow. The residues of conserved motifs are represented in sticks and colored in light purple. The sequence diagram of MtbPBP4 is also denoted in the lower part. The signal peptide region (Met1- Ala28) is colored in orange. (**b**) Structures of PBP4 homologs. The domain II region is located between part I and part II of domain I. Domains I, II, and III are colored in light green, light yellow, and gray, respectively.

To obtain structural insights into the role of MtbPBP4, a 3D model of MtbPBP4 was constructed using ColabFold: AlphaFold2 with the assistance of MMseqs2 [32] (pLDDT confidence 92.41). The N-terminal signal peptides (Met1–Ala28) and a long unstructured loop were excluded during the modeling process. As a result, the predicted region aligns with the region that was cloned and purified. The overall structure of MtbPBP4 consists of 11 α-helices and 10 β-sheets (Figure 1). The structure of MtbPBP4 can be primarily divided into two domains: domain I (part I: Pro67–Asp127, part II: Leu284–Thr461) and domain II (Asp128–Pro283) (Figure 3a). Domain I, also known as the penicillin-binding (PB) domain, is composed of a five-β-stranded sheet sandwiched between eight α-helices. On the other hand, domain II contains the topology of half a Rossmann fold, comprising a four-β-stranded sheet and two α-helices. Interestingly, domain II is inserted between domain I (part I) at the N-terminus and domain I (part II) at the C-terminus, creating a unique structural arrangement.

**Figure 4:**
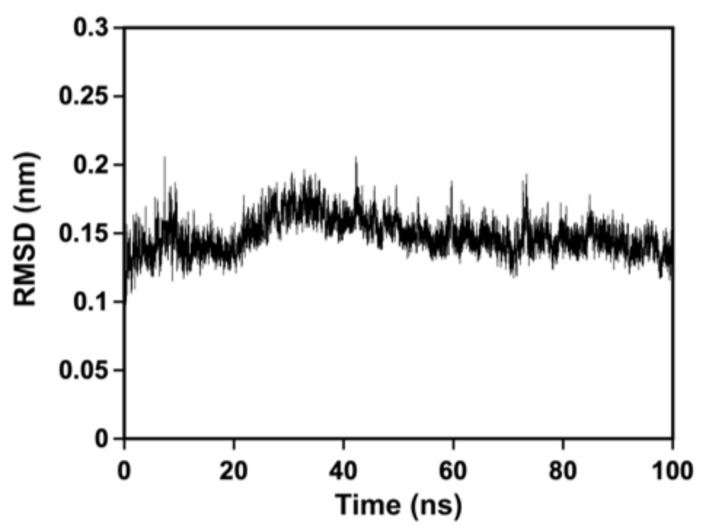
Structural dynamics analysis of MtbPBP4. The RMSD values of the Cα atoms from MtbPBP4 are plotted from 100 ns of MD simulations.

To assess the stability and flexibility of the predicted structure, MD simulations were performed. The RMSD of the Cα atoms was monitored over the course of 100 ns of simulation (Figure 4). The Cα RMSD values remained relatively constant, fluctuating between 0.1 and 0.2 nm (approximately 0.15 nm), indicating the structural stability of MtbPBP4 during the simulation. This result suggests that MtbPBP4 maintained its overall structure and did not undergo significant conformational changes throughout the simulation, indicating its stability under the simulated conditions.

According to the Dali search results [33], the overall fold of MtbPBP4 exhibits similarity to the structures of PBP4 homologs, with Z-scores exceeding 37 (Table 1). The primary structural difference between MtbPBP4 and its homologs lies in the presence of an additional domain, referred to as domain III. In the homologs of MtbPBP4, an extra domain III, consisting of approximately 70 additional amino acids, can be found between domain II, resembling the arrangement of Matryoshka (Russian dolls) (Figure 3b). However, despite the presence of domain III, domains I and II still exhibit a good superimposition between MtbPBP4 and its homologs (Table 1).

### 2.4. Active Site Cleft of MtbPBP4

**Figure 5:**
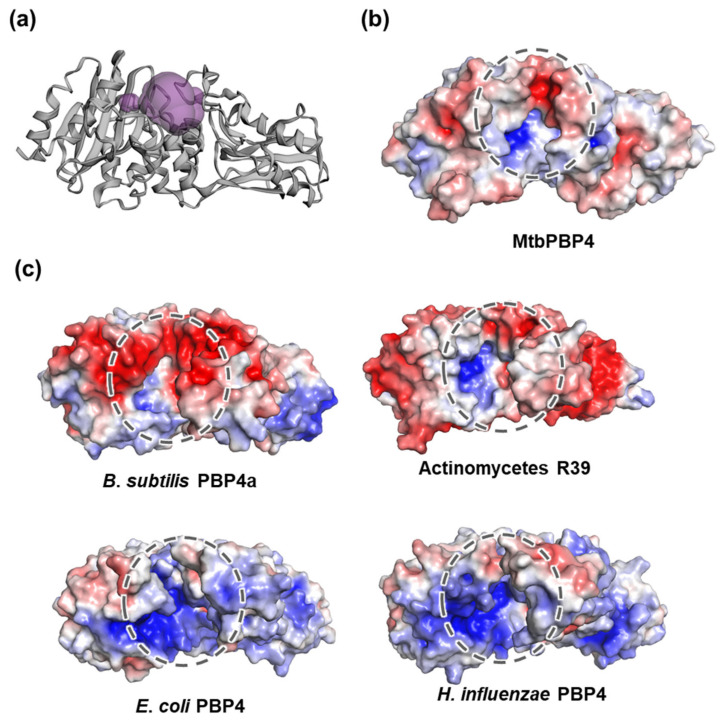
Putative active sites of MtbPBP4 and surface representations. (**a**) The predicted binding pocket of MtbPBP4 is represented in purple spheres. The ESP surface of (**b**) MtbPBP4 and (**c**) PBP4 homologs. Electrostatic surface potential represents different charge distributions on active sites marked as dotted circles. The negative and positive charges are colored in red and blue, respectively.

Our analysis of the putative active site in MtbPBP4, conducted using CASTp [34], identified a cleft in the binding pocket with a volume of 468.099 Å^3^ and a surface area of 400.493 Å^2^. This binding pocket is located between domains I and II (Figure 5a) and includes functionally important conserved residues such as Ser114, Ser295, Asp296, Asn297, and Thr409. The electrostatic potential (ESP) surface of MtbPBP4 displays a different charge distribution when compared to its homologs (Figure 5b,c). Notably, the presence of the additional β-barrel domain III results in varying pocket sizes among PBP4s. These differences between MtbPBP4 and its homologs may contribute to substrate specificity, possibly affecting the interactions with ligands or substrates at the binding pocket.

**Figure 6:**
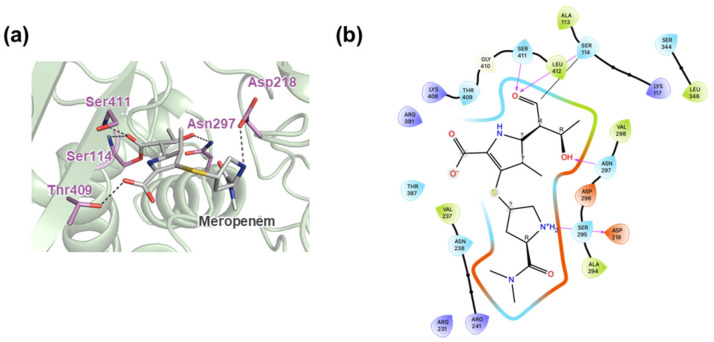
Docking simulation results for MtbPBP4 and a meropenem. (**a**) Covalent binding of a meropenem to MtbPBP4 through Ser114. The residues of MtbPBP4 that make hydrogen bond to a meropenem are represented by light purple sticks, and meropenem is colored in gray. Hydrogen bonds are represented by black dashes. (**b**) Detailed interaction residue information. Hydrogen bonds are represented by pink arrows, and covalent bonds are shown via the black lines. This figure was produced using Maestro of Schrödinger Suite (Schrödinger, LLC).

Previous results have indicated that MtPBP4 functions as a carboxypeptidase, cleaving the N-terminal D-alanine from mycobacterial peptidoglycan [34]. Other D,D-carboxypeptidases identified in *M. tuberculosis* include DacB1 and DacB2, and the carboxypeptidase activity of DacB2 has been shown to be inhibited by meropenem, a β-lactam antibiotic targeting PBPs in bacteria [35]. It has been shown in a previous study that MtbPBP4 binds to meropenem [36]. To further analyze the potential for designing inhibitors for MtbPBP4, docking simulations of meropenem and MtbPBP4 were performed using CovDOCK in Glide (Schrödinger, LLC., New York, NY, USA) (Figure 6a,b). The docking results showed that meropenem forms a covalent bond with Ser114 from the first motif STNK, with a docking score of −5.596 kcal/mol. Additionally, meropenem forms hydrogen bonds with Asp218, Asn297, Ser411, and Leu412. These structural insights into the interactions between meropenem and MtbPBP4 can guide the design of effective inhibitors targeting MtbPBP4.

Our study provides structural and functional insights into MtbPBP4, a critical enzyme involved in peptidoglycan synthesis in *M. tuberculosis*. By understanding the mode of action of β-lactam antibiotics such as meropenem on MtbPBP4, we can use this knowledge to guide the design of targeted inhibitors that specifically disrupt the enzyme’s activity. With the identification of the key residues involved in the binding and inhibition, we can focus on developing novel anti-tuberculosis agents that effectively target MtbPBP4, offering a promising vision for the production of new and potent drugs to combat MDR and XDR-TB.

## 3. Materials and Methods

### 3.1. Cloning, Expression, and Purification of MtbPBP4

The Rv3627c gene coding MtbPBP4 (NCBI reference sequence: WP_003899610.1) from Ala69 to Thr461 was amplified from the genomic DNA of *M. tuberculosis* H37Rv strain by polymerase chain reaction using two primers: (forward) 5′-CCA GGG AGC AGC CTC G GCC GCT GGC GTG ACC GCG GCG CT-3′ and (reverse) 5′-GCA AAG CAC CGG CCT CGT CA CGT CGT GCA CCC GCA GAA C-3′. The amplified gene was inserted into a pLIC-His vector for ligase-independent cloning (LIC) as previously described [37,38] This resulted in the incorporation of an N-terminal, 6xHis-tag, a TEV cutting site (ENLYFQ), and GAAAS residues for LIC cloning. The MtbPBP4-containing plasmid was transformed into *Escherichia coli* DH5α competent cells for cloning.

To overexpress MtbPBP4, cloned genes were transformed into *E*. *coli* BL21(DE3) competent cells. The transformed cells were grown in Luria broth with ampicillin until OD500 reached 0.6 at 37 °C, and protein overexpression was induced by 0.5 mM isopropyl-β-D-1-thiogalactopyranoside (IPTG). After further incubation in 15 °C for 20 h, the cells were harvested at 5000 rpm. The cell pellets were suspended in lysis buffer containing 20 mM Tris-HCl, pH 8.0, 500 mM NaCl, and 10% *v*/*v* glycerol and homogenized using ultrasonication. The resulting lysate was centrifuged at 13,000 rpm for 1 h to remove cellular debris. The supernatant was applied to a Ni–nitrilotriacetic acid (Ni-NTA) column for affinity chromatography, and the recombinant proteins were eluted by a stepwise increase in imidazole concentration from 0.1 M to 0.5 M. Subsequently, the protein was further purified using size-exclusion chromatography (SEC) on a Superdex 200 Increase 10/300 GL column (Cytiva) in a buffer containing 20 mM HEPES, pH 7.5, and 300 mM NaCl. The monomeric elution fractions were concentrated using a Vivaspin Turbo15 (Sartorius, Goettingen, Germany) with a 30 K molecular weight cutoff.

### 3.2. Oligomeric State Determination of MtbPBP4

The oligomeric state of MtbPBP4 was determined through gel filtration chromatography using a Superdex 200 Increase 10/300 GL column (Cytiva). The sample was run at a flow rate of 0.5 mL/min in a buffer containing 20 mM HEPES, pH 7.5, and 300 mM NaCl. To estimate the apparent molecular weight of MtbPBP4, we used a calibration curve based on standard proteins with known molecular weights. This involved comparing the elution volume of the target protein to that of the standard proteins. The gel filtration calibration kit LMW (Cytiva) included ribonuclease A (13.7 kDa), carbonic anhydrase (29 kDa), ovalbumin (43 kDa), conalbumin (75 kDa), and blue dextran 2000. To determine the oligomeric states of the MtbPBP4 with quantitative values, we conducted a SEC-MALS experiment using a fast protein liquid chromatography (FPLC) system (Cytiva) connected to a MiniDAWN TREOS MALS instrument (Wyatt, Santa Barbara, CA, USA). A Superdex 200 Increase 10/300 GL (Cytiva) gel filtration column was pre-equilibrated with a buffer containing 20 mM HEPES, pH 7.5, and 300 mM NaCl. The detector was normalized using 2 mg/mL bovine serum albumin (Thermo Fisher Scientific, Waltham, MA, USA), and 100 μL of the protein solution was injected at a concentration of 3.5 mg/mL. The acquired data were analyzed using ASTRA 8 software (Wyatt). Chromatography was performed on a Superdex 200 Increase 10/300 GL column with a buffer containing 20 mM HEPES, pH 7.5, and 300 mM NaCl at a flow rate of 0.5 mL/min.

### 3.3. Structure Prediction of MtbPBP4

For the prediction of MtbPBP4, we employed ColabFold, which is a user-friendly and fast implementation of AlphaFold2 [32]. The protein sequence of Rv3627c from *M. tuberculosis* H37Rv (UNIPROT ID O06380) served as the input sequence, and a Multiple Sequence Alignment (MSA) was generated using MMseqs2. The calculations were run using the ColabFold online platform (AlphaFold2.ipynb) with default options. To visualize the predicted structures, we utilized the *PyMOL* program (PyMOL Molecular Graphics System, version 2.5.3; Schrödinger, LLC.).

### 3.4. MD Simulation

Molecular Dynamics (MD) simulations of MtbPBP4 were conducted using the GROMACS 2023.1 packages [39]. The AMBER99SB-ILDN force field was selected for the simulations. The molecules were placed in a cubic box filled with TIP3P water molecules and neutralized by adding Na^+^ and Cl^−^ ions. Steepest descent minimization was performed to optimize the system. Subsequently, equilibration steps were carried out in both the NVT ensemble (constant number of particles, volume, and temperature) and NPT ensemble (constant number of particles, pressure, and temperature) for 100 ps at 1 atm and 300 K. The V-rescale thermostat and Parrinello-Rahman barostat were used for controlling temperature and pressure in the NVT and NPT ensembles, respectively. After equilibration, MD simulations of the predicted MtbPBP4 were performed for 100 ns at 300 K utilizing the linear constraint solver (LINCS) algorithm for constraining bond lengths and the particle mesh Ewald (PME) method for handling long-range electrostatic interactions. The resulting MD trajectories were analyzed using GROMACS distribution programs, particularly gmx_rms for calculating parameter values such as root mean square deviation (RMSD). The analysis was processed using the XMGrace program (Oregon Graduate Institute of Science and Technology, Hillsboro, OR, USA), and plots were generated using KaleidaGraph 5.0 software (Synergy Software, Reading, PA, USA).

### 3.5. Covalent Docking

The MtbPBP4 protein was prepared for Glide docking calculations using the Protein Preparation Wizard of Schrödinger Suite (Schrödinger, LLC., New York, NY, USA). Proper protein preparation is crucial for accurate protein–ligand docking simulations. The active site of the protein was identified, and key residues such as Ser114, Ser295, Asn297, and Thr409 were selected for further molecular docking analysis. To prepare the protein, the atoms of the protein were scaled with a van der Waal’s radius at a factor of 1 Å, and a partial charge cutoff of 0.25 Å was applied. The ligand structure of meropenem was obtained from the RCSB PDB (http://www.rcsb.org accessed on 6 January 2024) and prepared using LigPrep (Schrödinger, LLC). The stereoisomers’ chirality was determined from the 3D structure, and all other parameters were kept as default during the LigPrep preparation. For covalent docking, the CovDock module (Schrödinger, LLC) was used. The amino acid Ser114 was set as the reactive residue, which would have a nucleophilic interaction with the O− and carbonyl group of meropenem acting as the electrophile. The reaction type was predefined as Beta Lactam Addition using the pre-installed options in CovDock [40].

## 4. Conclusions

Our comparison of MtbPBP4 sequences indicated that this enzyme belongs to the peptidase family S13, specifically D-Ala-D-Ala carboxypeptidase C. From SEC experiments, it was confirmed that MtbPBP4 mainly exists in a monomer state. The predicted three-dimensional structure of MtbPBP4 is similar to that of the known PBP4 homologs, but certain differences were identified, such as the absence of domain III, which is typically inserted between domain II in homologs. MtbPBP4 does contain conserved active site motifs, consistent with other homologs. The docking of β-lactam antibiotics to MtbPBP4 revealed the binding site located in these conserved residues. As a carboxypeptidase, MtbPBP4 is involved in the cleavage of peptidoglycan, which plays a crucial role in mycobacterial morphology and cell division. These structural insights provide information for the development of anti-tuberculosis drugs, as targeting MtbPBP4 may represent a promising approach to disrupting cell wall synthesis and inhibiting mycobacterial growth.

## Figures and Tables

**Table 1:** Comparison of the homologs of PBP4.

Organism	PDB Code	Chain	No. of Cα Aligned	RMSD (Å)	Z-Score	% Identity
*B*. *subtilis*	1W5D	A	345 (361/458:DALI)	1.370 (1.8: DALI)	40.8	27
Actinomycetes	1W79	A	335 (363/466: DALI)	1.190 (1.8:DALI)	40.9	30
*E*. *coli*	2EX2	A	330 (355/456:DALI)	2.518 (2.3:DALI)	37.7	28
*H. influenzae*	3A3D	A	326 (354/453:DALI)	1.250 (2.2: DALI)	37.8	26

## Data Availability

Data contained within the article.
